# CD8^+^ T cells promote ZIKV clearance and mitigate testicular damage in mice

**DOI:** 10.1038/s44298-024-00033-5

**Published:** 2024-06-17

**Authors:** Rafael K. Campos, Yuejin Liang, Sasha R. Azar, Judy Ly, Vidyleison Neves Camargos, E. Eldridge Hager-Soto, Eduardo Eyzaguirre, Jiaren Sun, Shannan L. Rossi

**Affiliations:** 1 Department of Microbiology and Immunology, University of Texas Medical Branch, Galveston, TX USA; 2 Department of Pathology, University of Texas Medical Branch, Galveston, TX USA; 3 Houston Methodist Research Institute, Houston, TX USA; 4 Institute for Human Infection and Immunity, University of Texas Medical Branch, Galveston, TX USA; 5 Center of Tropical Disease, University of Texas Medical Branch, Galveston, TX USA

**Keywords:** Microbiology, Virology, Viral transmission

## Abstract

Zika virus (ZIKV) causes human testicular inflammation and alterations in sperm parameters and causes testicular damage in mouse models. The involvement of individual immune cells in testicular damage is not fully understood. We detected virus in the testes of the interferon (IFN) α/β receptor^−/−^ A129 mice three weeks post-infection and found elevated chemokines in the testes, suggesting chronic inflammation and long-term infection play a role in testicular damage. In the testes, myeloid cells and CD4^+^ T cells were absent at 7 dpi but were present at 23 days post-infection (dpi), and CD8^+^ T cell infiltration started at 7 dpi. CD8^−/−^ mice with an antibody-depleted IFN response had a significant reduction in spermatogenesis, indicating that CD8^+^ T cells are essential to prevent testicular damage during long-term ZIKV infections. Our findings on the dynamics of testicular immune cells and the importance of CD8^+^ T cells function as a framework to understand mechanisms underlying observed inflammation and sperm alterations in humans.

## Introduction

Zika virus (ZIKV) is a positive-strand RNA virus of the *Flaviviridae* family that was discovered in Uganda in 1947 and is transmitted primarily by mosquito bites. However, Foy and colleagues proposed an additional mode of transmission, sexual transmission, when a likely case was identified in the United States in 2008–2009, involving a man that had returned from Senegal in 2008-2009^1,2^. Sexual transmission was confirmed during the 2015–2017 ZIKV epidemics^3^, which together with animal experiments^4^, confirmed that ZIKV could be sexually transmitted^1,3,5–7^. Confirmation of this mode of transmission raised numerous concerns with human health implications. These include effects on ZIKV circulation during epidemics, enhanced concerns of ZIKV congenital syndrome, and testicular damage. ZIKV infectious particles or ZIKV RNA were present in semen and vaginal secretions long after the initial infection^8,9^. In semen, ZIKV infectious particles were detected for up to 69 days^10^, and ZIKV RNA has been detected for up to 370 days after initial infection^11^. Men that shed ZIKV RNA for long periods have signs of male reproductive tract inflammation, with higher leukocyte counts^12^, elevated cytokine levels^12^, and several alterations in sperm^13^. Since ZIKV does not commonly cause fatal disease, acquiring tissue samples of people recently infected with ZIKV is challenging.

Using animal and cell culture models, components of the male reproductive tract shown to be a site of long-term replication and inflammation include the epididymides^14–16^, the prostate^17,18^, and the testes^19–21^, leading to reduced fertility in mouse models^21^. Studies using ZIKV-infected non-human primates^22^ and mouse models^20,21^ have also shown that ZIKV infection can lead to histopathologic lesions in the testes, which is a health concern for infected men in addition to impacting fertility. The testes are the site of spermatogenesis and may play a key role in ZIKV-caused infertility as well as viral maintenance for sexual transmission. Studies have shown that ZIKV can establish long-term infections in Sertoli cells, germ cells^23^, and spermatogonia^23^.

The testes are an immune-privileged organ with a delicate balance maintained by the blood-testis barrier (BTB) and regulatory, tolerogenic, immune cells, including macrophages, dendritic cells, mast cells, cluster of differentiation (CD)4^+^ and CD8^+^ T cells^24,25^. Macrophages are the major population of cells in the testes, representing approximately 20% of cells in the interstitial space under physiological conditions^25^. Macrophages and dendritic cells have been found to be important targets of ZIKV visceral replication^26^. In addition to immune cells native to the testes, cell infiltration, and inflammation have also been shown to be triggered by ZIKV infection in animal models^20,27^, and one cell population confirmed to invade the testes is CD8^+^ T cells^28^. Macrophages and dendritic cells were implicated as important initial targets of replication which may help the virus invade the testes^26,29^. However, the dynamics of infiltration of immune cells in the testes and their roles in clearing ZIKV infection are not well understood.

Using mouse models, we have investigated cytokine levels and immune cells involved in the response against ZIKV long-term testicular infection and the role of CD8^+^ T cells in clearing ZIKV from testicular tissue and reducing the odds of testicular damage.

## Results

### ZIKV long-term infection of the testes in human primary Sertoli cells and mouse models

**Fig. 1:**
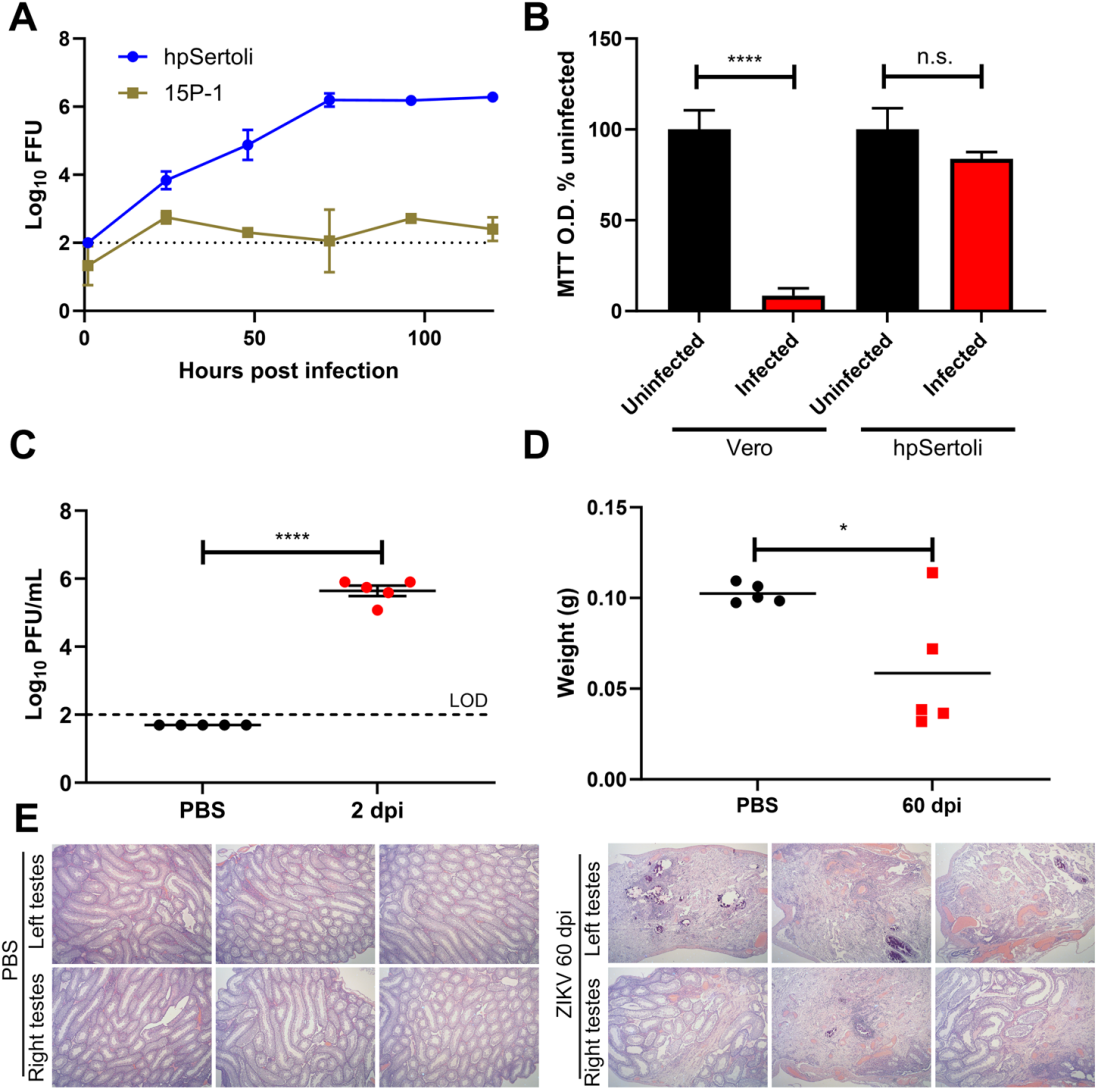
Human primary Sertoli cells support long-term infection of ZIKV with minimal cell death, but widespread damage is observed in testes of A129 mice. **A** ZIKV PRVABC59 growth curve on human primary Sertoli (hpSertoli) cells or mouse Sertoli (15P-1) cell line at an MOI of 0.1, the supernatant was collected at each time point and titrated. **B** Sertoli cells show minimal cell death when MTT assay is used as a proxy for cell viability. For **A**, **B**, bars show mean and error bars represent standard deviation. Statistical significance was assessed with one-way ANOVA with Sidak’s multiple comparison correction. **C** Viremia in A129 mice at 2 dpi after mice were infected intraperitoneally with a target dose of 3log_10_ PFU. **D** A129 mice testicular weight 60 dpi with ZIKV. Statistical significance was assessed using a two-tailed t-test. Lines represent the mean. **E** Histopathological damage observed in mice testes 60 dpi with ZIKV. In the experiment related to figures **C**–**E**, 5 mice were used per condition. *p < 0.05; ****p < 0.0001.

Sertoli cells comprise the BTB and are essential for maintaining seminiferous tubule (ST) architecture. Both murine Sertoli cell line model 15P-1 and human primary (hp) Sertoli cells support ZIKV PRVABC59 infection (Fig. 1A). hpSertoli cells support higher viral titers in the supernatant (6 log_10_ plaque-forming units (PFU)/ml). In both of these cell types, viral titers were maintained for at least 5 days while no substantial cell death was observed. For hpSertoli cells, we measured that infection at a multiplicity of infection (MOI) of 0.1 for 5 days did not significantly lower cell metabolic activity (Fig. 1B). We then performed in vivo experiments using the A129 mouse model, infecting these mice with a target dose of 3 log_10_ PFU of ZIKV intraperitoneally. Viremia at 2 dpi confirmed that all animals were infected (Fig. 1C). The weight of the testes was significantly reduced compared to that of the PBS-injected mice (Fig. 1D). Several testes from infected mice had apparent testicular damage with diffuse tubular necrosis upon histological examinations (Fig. 1E). This appears to contrast with the resistance to cell death upon infection observed in hpSertoli cells in culture (Fig. 1A, B). It is possible that the immune system contributes to the testicular damage by disrupting the testicular microenvironment.

### ZIKV long-term infection of the testes in the A129 mouse model induces local cytokine and chemokine expression

**Fig. 2:**
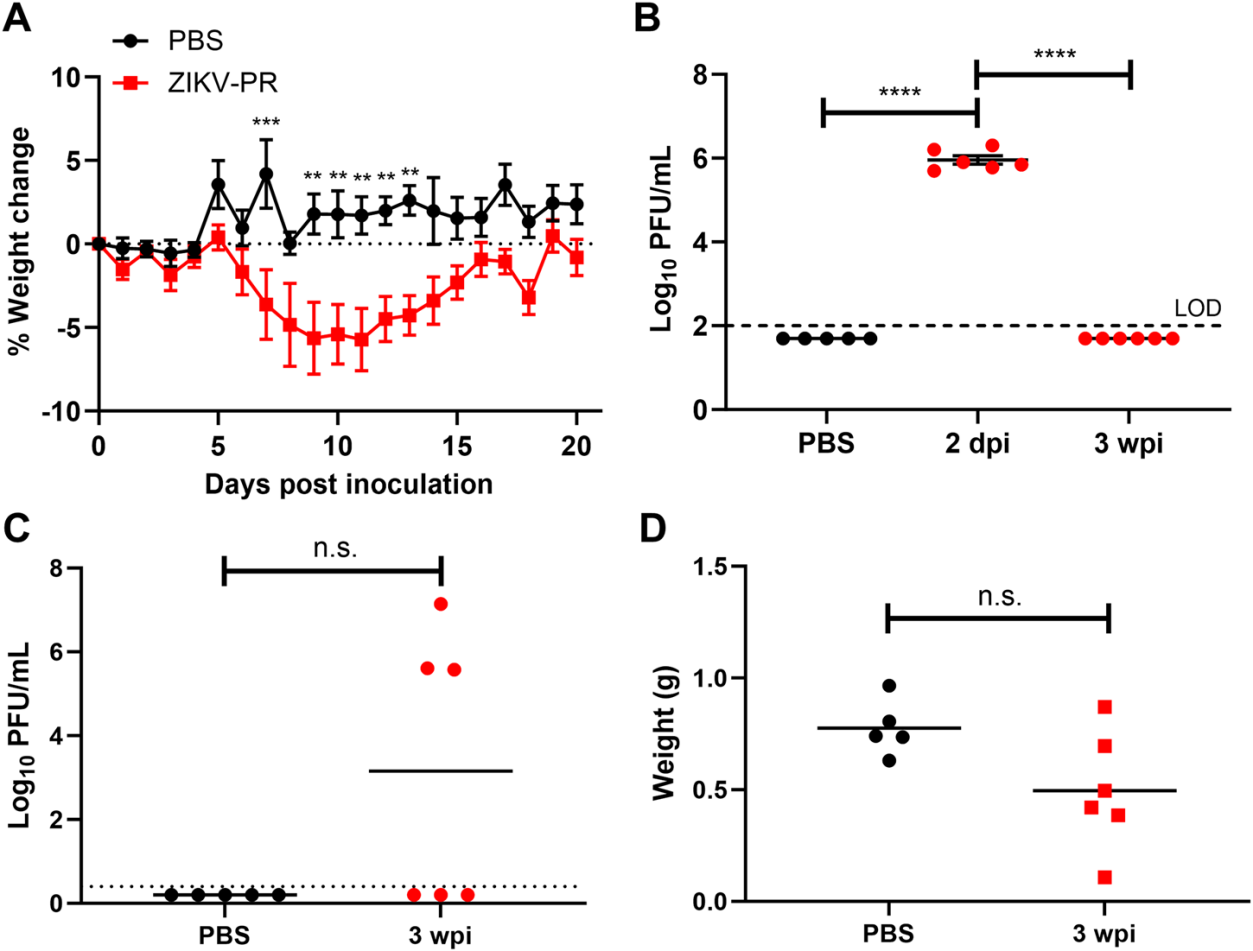
ZIKV replication in testes of A129 mice after clearance of systemic infection. **A** Weights of A129 mice infected with ZIKV PRVABC59. Mice were infected intraperitoneally with a target dose of 3log_10_ PFU. Statistical significance was assessed with two-way ANOVA with Tukey’s multiple comparison correction. Error bars represent standard error of the mean. **B** Virus detection in the plasma of A129 mice, uninfected, or at 2 dpi or 3 wpi. Lines represent the mean. **C** Viral titer in the testes at 3 wpi. **D** Testicular weight of the mice at 3 wpi. Bars show mean. In the experiment related to this figure, 5 mice were used in the PBS group, and 6 in the infection group. Horizontal dotted lines represent the limit of detection. **p < 0.01; ***p < 0.001; ****p < 0.0001.

**Fig. 3:**
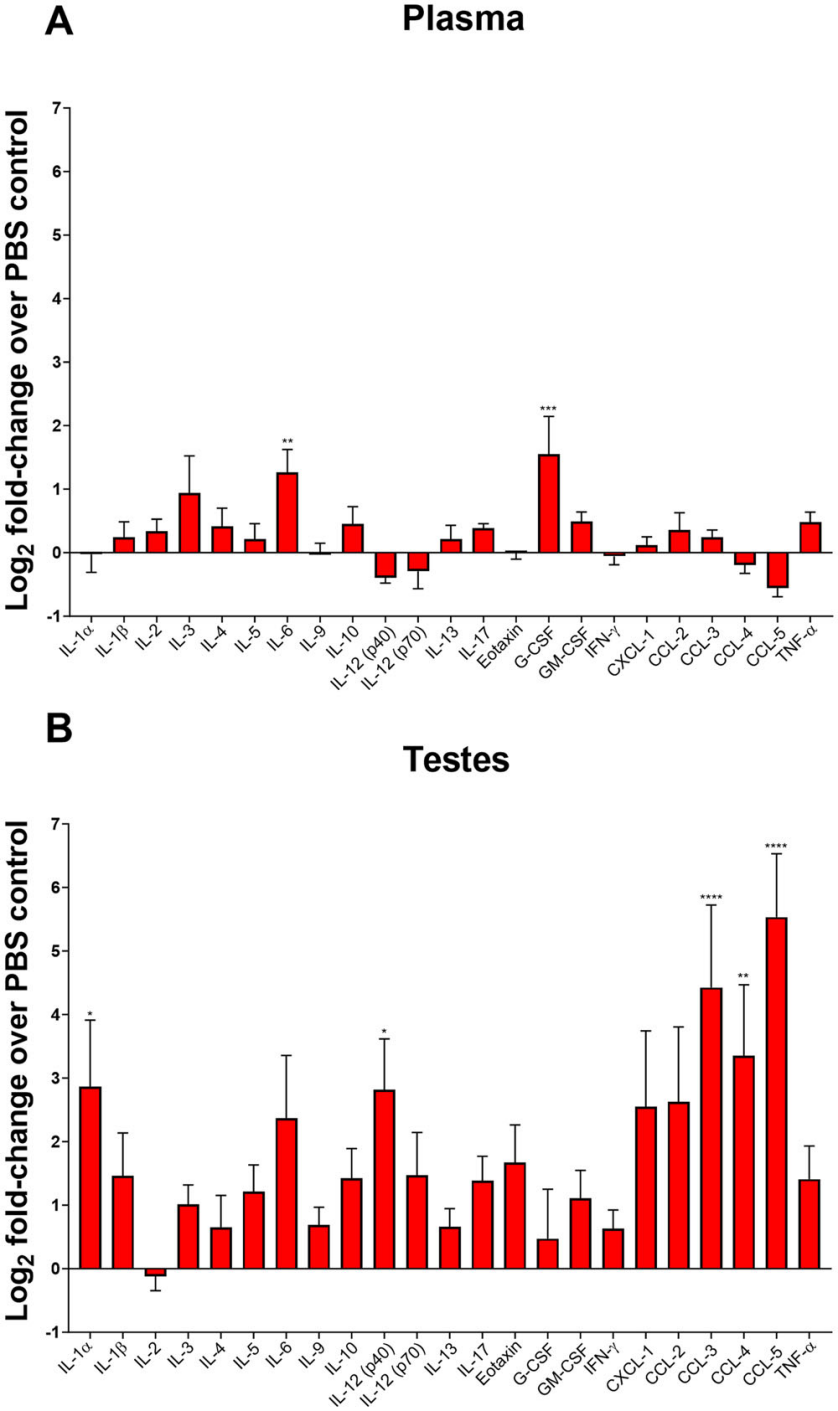
Long-term ZIKV infection of A129 mice induces local activation of cytokines and chemokines. **A** Log_2_ of the fold-change of cytokines and chemokines measured in the plasma of ZIKV-infected mice. **B** Log_2_ fold-change induction of cytokines and chemokines in the testes of ZIKV-infected mice. The data shown in this figure are from the same mouse groups as in Fig. 2. Statistical significance was assessed with one-way ANOVA with Sidak’s multiple comparison correction in between PBS control groups (*N* = 5) and the infected groups (*N* = 6), for each cytokine and chemokine. Bars show mean and error bars represent standard error of the mean. *p < 0.05; **p < 0.01; ***p < 0.001; ****p < 0.0001.

To investigate whether long-term ZIKV infection of the testes induced local immune system activity, we infected A129 mice with a target dose of 3 log_10_ PFU of ZIKV. We observed that the mice showed acute signs of disease, such as lethargy, ruffled fur, and had significant body weight loss, with peak weight loss at 10 days post-infection (dpi) before recovery (Fig. 2A). All mice developed viremia at 2 dpi with an average around 6 log_10_ PFU/ml, whereas none had detectable viremia three weeks post-infection (Fig. 2B), suggesting systemic infection had been cleared. In contrast, at 21 dpi we detected viable virus in the testes of 3 out of 6 mice, with 2 of these mice having virus in both of the testes, and the highest titer detected being 7 log_10_ PFU/mg of tissue (Fig. 2C). Although some infected mice had testes that were substantially lighter than mice in the PBS group, the testicular weight difference between these groups were not statistically significant (Fig. 2D). Cytokines and chemokines presented in testicular macerates and plasma at this time were analyzed on a multiplex bioassay (BioPlex). We detected a significant but moderate (< 5-fold) increase in interleukin (IL)-3 and G-CSF in the plasma (Fig. 3A). In testicular homogenates, we detected a significant and drastic change in the pro-inflammatory cytokines IL-1α and IL-12(p40), and in the chemokines C-C motif chemokine ligand (CCL)-3, CCL-4 and CCL-5 (Fig. 3B). The elevated levels of chemokines suggest that immune cells are being recruited in the testes, and chronic inflammation may occur three weeks after the initial infection.

### Myeloid cells are present in the lymphoid organs primarily at 7 dpi and in the testes at 23 dpi

**Fig. 4:**
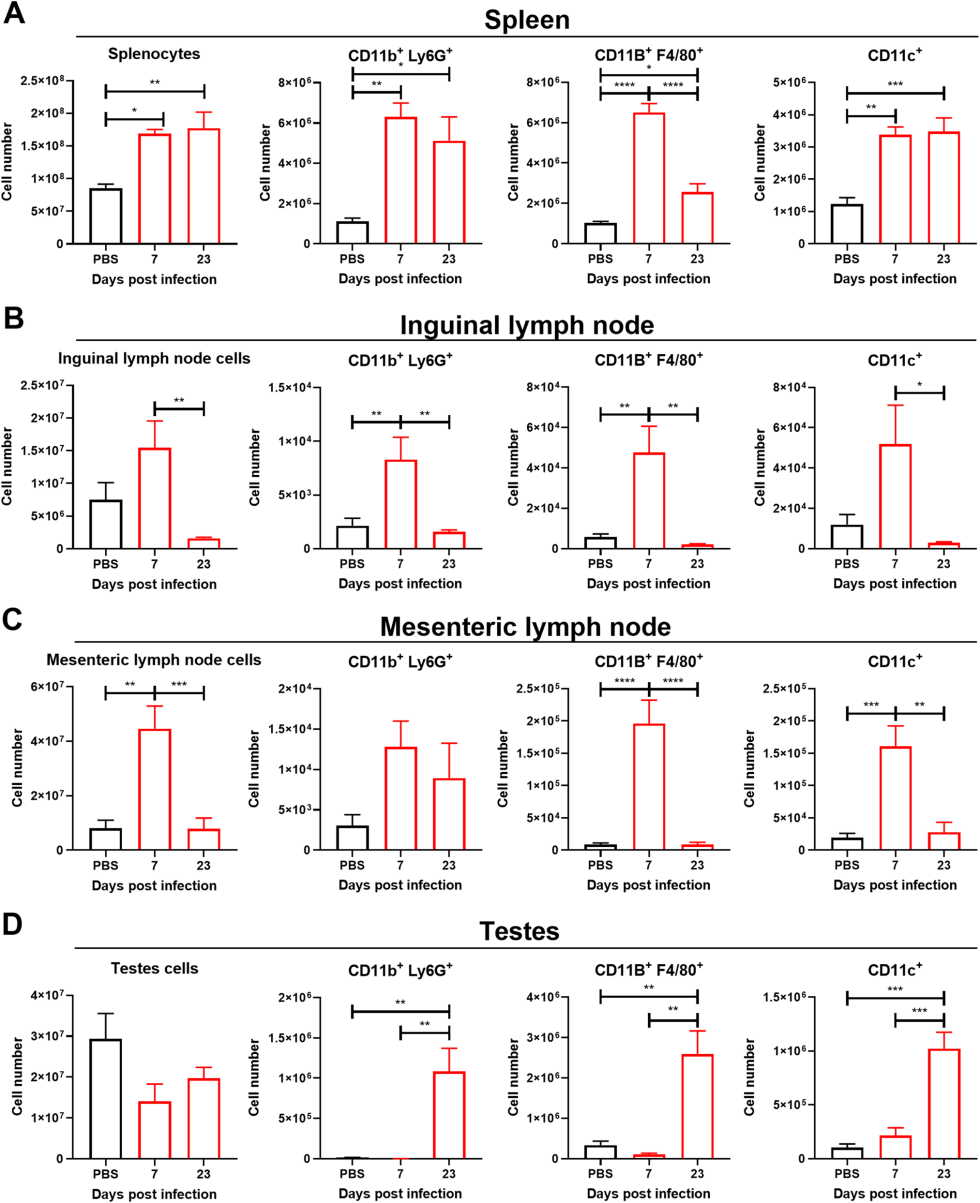
Macrophages, neutrophils and DCs infiltrate testes at 23 dpi. Cell count and myeloid cells for **A** the spleen, **B** the inguinal lymph nodes, **C** the mesenteric lymph nodes and **D** testes. Mice were infected intraperitoneally with a target dose of 3log_10_ PFU. In the experiment related to this figure, 5 mice were used for the PBS group, 5 mice for the 7 dpi group, and 6 mice for the 23 dpi group. Red bars show ZIKV-infected mice, and black bars denote uninfected baseline controls. Statistical significance was assessed with one-way ANOVA with Sidak’s multiple comparison correction. Comparisons were made between each of the groups and only significant results are shown. Bars show mean and error bars represent standard error of the mean. *p < 0.05; **p < 0.01; ***p < 0.001; ****p < 0.0001.

To investigate immune cell populations in the context of long-term ZIKV infection of the testes, we infected A129 mice with a target dose of 3 log_10_ PFU of ZIKV intraperitoneally. We observed similar weight loss, disease, and recovery of visible disease signs as in the previous experiment. In addition to collecting testicular tissue, to assess the systemic presence of immune cells in the different lymphoid organs, we collected the spleen, the mesenteric lymph nodes, and the inguinal lymph nodes. Samples were processed for flow cytometry, and data were collected on immune cell populations at 7 and 23 dpi. We counted the total number of cells in the testes and the lymphoid organs and found that the spleen and the mesenteric lymph nodes had increased total cell counts at 7 dpi. Cells from the lymph nodes were significantly reduced from 7 dpi to 23 dpi, but total cells in the spleen remained elevated at the 23 dpi timepoint (Fig. 4A–C). In contrast to the lymphoid organs, the total number of cells in the testes was not significantly different (Fig. 4D). In the spleen, the mesenteric and inguinal lymph nodes, there was an increase in macrophages and dendritic cells at 7 dpi, compared to the organs of PBS-inoculated controls (Fig. 4A–C). Neutrophils were also elevated in the spleen and the inguinal lymph nodes at 7 dpi but did not reach statistical significance in the mesenteric lymph nodes (Fig. 4A–C). At 23 dpi, the levels of these cells were either similar to 7 dpi or decreased in the lymphoid organs (Fig. 4A–C). In contrast to the lymphoid organs, myeloid cells were not present in the testes at 7 dpi but were highly increased at 23 dpi (Fig. 4D). An increase in the number of myeloid cells in the testes late in infection indicates chronic inflammation.

### CD4^+^ T cells but not CD8^+^ T cells recruitment to the testes is delayed in comparison to the lymphoid organs

**Fig. 5:**
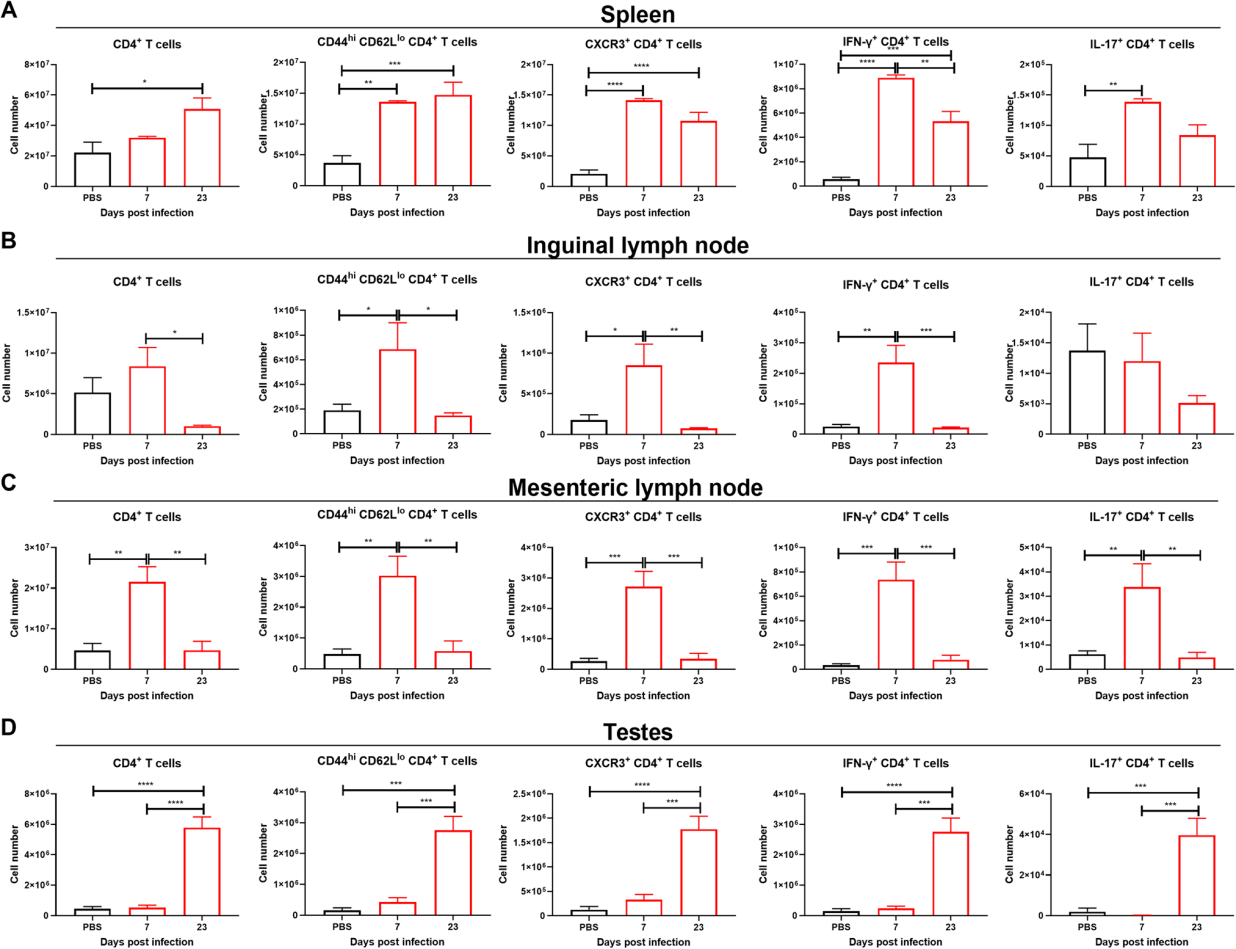
CD4^+^ T cells accumulate in testes at 23 dpi. CD4^+^ T cells for **A** the spleen, **B** the inguinal lymph nodes, **C** the mesenteric lymph nodes and **D** testes. Red bars show ZIKV-infected mice, black bars denote uninfected baseline controls. The data shown in this figure are from the same mouse groups as in Fig. 4. Statistical significance was assessed with one-way ANOVA with Sidak’s multiple comparison correction. Comparisons were made in between each of the groups and only significant results are shown. Bars show the mean and error bars represent the standard error of the mean. *p < 0.05; **p < 0.01; ***p < 0.001; ****p < 0.0001.

As T cells are an integral part of the immune response against ZIKV, we assessed CD4^+^ T cells at 7 and 23 dpi in these ZIKV-infected mice. We found that CD4^+^ effector T cells (CD44^hi^, CD62L^lo^) were increased in the lymphoid organs at 7 dpi, and then decreased back to original levels at 23 dpi in the inguinal and mesenteric lymph nodes but not in the spleen (Fig. 5A–C). In contrast, CD4^+^ effector T cells were not significantly increased in the testes at 7 dpi but were increased at 23 dpi (Fig. 5D). Effector T cells prominently express CXCR3, which plays a crucial role in regulating T cell trafficking and function^30,31^. In the lymphoid organs, CD4^+^ CXCR3^+^ T cells were increased at 7 dpi, and decreased again in the lymph nodes at 23 dpi, but remained elevated in the spleen (Fig. 5A–C). In the testes, CD4^+^ CXCR3^+^ T cells were not increased at 7 dpi, but were drastically increased at 23 dpi, similar to what was observed for myeloid cells. IFN-γ plays a chief role in virus immunity and countering ZIKV infections. We evaluated CD4^+^ IFN-γ^+^ T cells and found that it was broadly consistent with the presence of CD4^+^ effector T cells, being increased in the lymphoid organs but not in the testes at 7 dpi (Fig. 5A–D). At 23 dpi, CD4^+^ IFN- γ^+^ T cells were decreased in the lymphoid organs in comparison to 7 dpi (Fig. 5A–C), but were strongly increased in the testes, suggesting a chronic activation of the IFN antiviral response in testicular tissues. IL-17 is an important factor related to allergies and autoimmunity, and functions in many biological processes, including chronic inflammation. IL-17 is thought to be produced mainly by Th17 cells, a subset of CD4^+^ T cells. We found that CD4^+^ IL-17^+^ T cells were increased at 7 dpi in the spleen and the mesenteric lymph nodes but not in the testes and the inguinal lymph nodes, which drain from the testes (Fig. 5A–D). At 23 dpi, CD4^+^ IL-17^+^ T cells were no longer statistically increased compared to PBS controls, and the inguinal lymph nodes continued without any increase in these cells. In contrast, the testes had a large population of CD4^+^ IL-17^+^ T cells present at this time point. We also identified a population of possible memory cells CD4^+^ CD44^hi^ CD62L^hi^ which increased in the lymphoid organs at 7 dpi and in the testes at 23 dpi (Supplementary Fig. 2). This observation is likely not relevant for this primary infection but may be important if a secondary ZIKV infection were to occur.

**Fig. 6:**
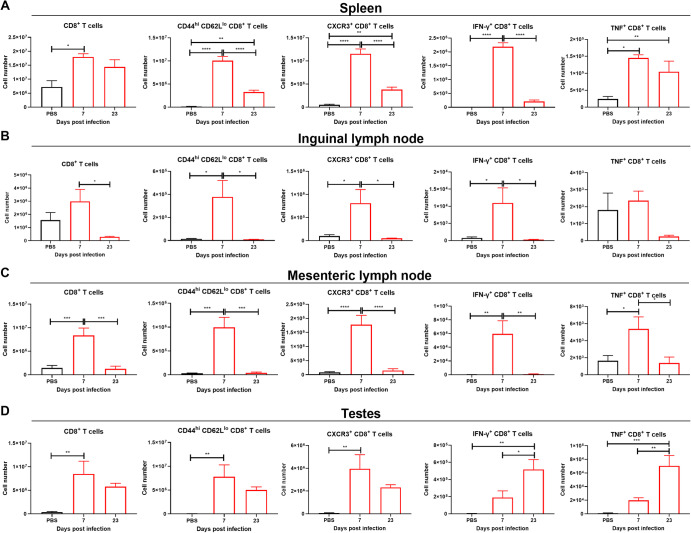
CD8^+^ T cells accumulate in testes at 7 and 23 dpi and in lymph nodes at 7 dpi. CD8^+^ T cells for **A** the spleen, **B** the inguinal lymph nodes, **C** the mesenteric lymph nodes, **D** testes. Red bars show ZIKV-infected mice, black bars denote uninfected baseline controls. The data shown in this figure are from the same mouse groups as in Fig. 4. Statistical significance was assessed with one-way ANOVA with Sidak’s multiple comparison correction. Comparisons were made in between each of the groups and only significant results are shown. Bars show mean and error bars represent standard error of the mean. *p < 0.05; **p < 0.01; ***p < 0.001; ****p < 0.0001.

In all organs tested, effector CD8^+^ T cells (CD44^hi^, CD62L^lo^) were increased at 7 dpi, earlier than other cell types tested (Fig. 6A–D). At 23 dpi, effector CD8^+^ T cells and CD8^+^ CXCR3^+^ T cells were decreased compared to 7 dpi in the lymphoid organs and were no longer significantly higher than in the PBS group in the testes (Fig. 6A–D). That was also the case for CD8^+^ IFN- γ^+^ T cells in the lymphoid tissues but not for the testes (Fig. 6A–D). In testicular tissue, CD8^+^ IFN-γ^+^ T cells were not significantly elevated at 7 dpi, whereas a significant increase was noted at 23 dpi (Fig. 6D). This suggests that effector CD8^+^ T cells were present at 7 dpi in the testes, but their ability to produce IFN-γ was limited. CD8^+^ T cells producing IFN-γ were only detected at 23 dpi. Expression of programmed Death-1 (PD-1) was observed in both CD4^+^ and CD8^+^ T cells. PD-1 is an inhibitory receptor involved in T cell exhaustion which is expressed upon activation of T cells (Supplementary Fig. 3).

### C57BL/6 J CD8^−/−^ have decreased spermatogenesis compared to wild type control mice

As the flow cytometry data indicated a critical role for T cells, we next sought to assess their role in the establishment of viral infection or viral testicular clearance, and if they influenced the severity of testicular damage in long-term ZIKV infection. Two mouse models, the A129 mice (IFNα/βR^−/−^) or the immunocompetent C57BL/6 J mice, were used; cohorts of mice were treated with anti-mouse CD3ε F(ab’)2 fragments or control f(ab’)2 fragments of polyclonal hamster IgG (5 injections of 100 µg each of anti-CD3 or control treatments on the following dpi: -3, -1, 2, 7, and 14). Because ZIKV cannot efficiently infect C57BL/6 J mice, these mice were additionally transiently injected with 1.5 mg/mouse of IFNAR-blockading antibody one day before infection to allow ZIKV to infect the mouse tissues and reach the testes. Mice were infected with ZIKV as previously done. To confirm the depletion of CD3^+^ cells, we measured CD3 in the blood by flow cytometry on 2 and 18 dpi (Supplementary Fig. 4). In A129 mice, the depletion of CD3^+^ cells was approximately 70% at 2 dpi, and >99% at 18 dpi, and in C57BL/6 J, the depletion of CD3^+^ cells was approximately 80% at both timepoints (Supplementary Fig. 4A, E). However, in both mouse models, no significant difference was observed in the percentage of ST exhibiting spermatogenesis (Supplementary Fig. 4B, C, F, G) or in testicular weights (Supplementary Fig. 4D, H) between infected control and CD3-depleted mice.

**Fig. 7:**
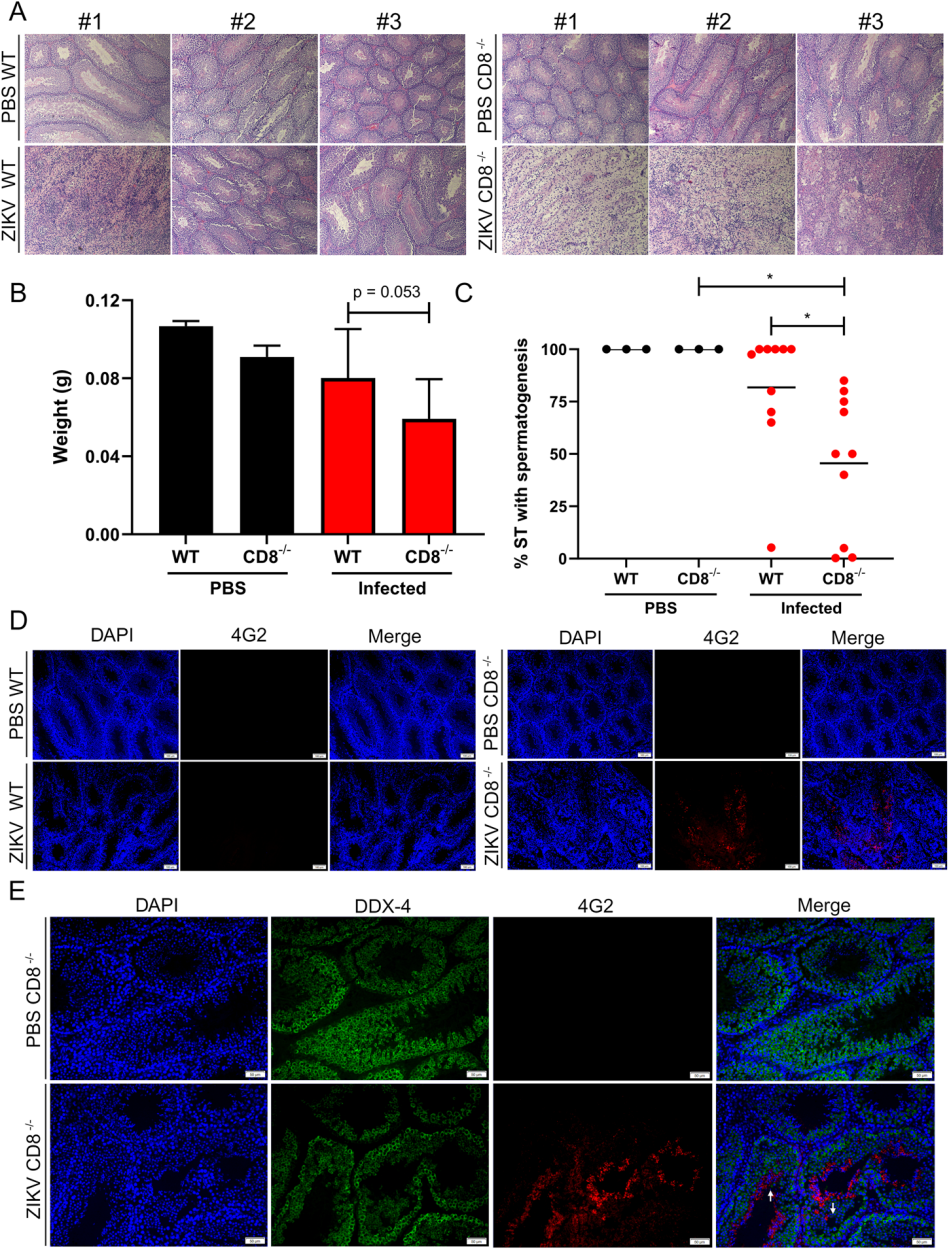
CD8^+^ T cells are important to prevent significant testicular damage upon long-term ZIKV infection in C57BL/6 J mice transiently treated with anti-IFN antibodies. **A** Histology of WT C57BL/6 J mice or CD8^−/−^ mice testes. Mice were infected intraperitoneally with a target dose of 5log_10_ PFU/mouse. In the experiment related to this figure, 3 mice were used for each of the PBS groups, and 10 mice were used for each of the CD8^−/−^ groups. **B** Testicular weight. Statistical significance was assessed with one-way ANOVA with Sidak’s multiple comparison correction in between WT and CD8^−/−^ conditions, either uninfected or infected. Bars show mean and error bars represent standard deviation. **C** Percentage of spermatogenesis quantified from the histology by a pathologist blinded to the samples. Statistical significance was assessed using one-way ANOVA with Sidak’s multiple comparison correction. Groups using WT mice were compared against groups using CD8^−/−^ mice only within the same treatment, and groups of mice injected with PBS were compared to groups of mice injected with ZIKV only for the same mouse type. Lines represent the mean. **D**, **E** Immunofluorescence of testicular samples showing cell nuclei (blue), ZIKV envelope protein (red), and DDX-4 (green). The brightness and contrast of these images were adjusted for optimal visibility. The white arrows show cell detachment from the wall of the ST. *p < 0.05.

As CD8^+^ T cells were the only cell type detected abundantly in the testes at 7dpi, we hypothesized that these cells might contribute to the early immune reaction observed, leading to the recruitment of other immune cells. To test that hypothesis, we intraperitoneally infected wild type (WT) C57BL/6 J mice or its CD8^−/−^ counterpart with a 5 log_10_ PFU/mouse target dose. In this experiment, to obtain a more permissive testicular damage phenotype, we injected the mice twice with 1.5 mg/mouse of IFNAR-blockading antibody one day before infection and five dpi. To quantify testicular damage in the hematoxylin-eosin-stained histology slides (Fig. 7A), a board-certified pathologist blinded to the samples quantified the percentage of the ST with spermatogenesis in each slide. The CD8^−/−^ mice had significantly decreased percentage of ST with spermatogenesis compared to the WT C57BL/6 J mice (Fig. 7A–C), suggesting the presence of CD8^+^ T cells is key to avoiding or reducing the severity of the testicular damage. We evaluated the slides by immunofluorescence (Fig. 7D) and detected ZIKV envelope protein in 0 out of 10 WT mice and 6 out of 10 CD8^−/−^ mice at 23 dpi, which was statistically significant by Fisher’s exact test (Supplementary Table). We observed the presence of viral envelope protein in cells positive for DDX-4 (marker of spermatogonia, spermatocytes, and round spermatids) and in cells negative for DDX-4, suggesting ZIKV infects various cell types in the ST (Fig. 7E).

## Discussion

ZIKV has caused large epidemics worldwide, but most notably across the Americas in 2015 and 2016. This epidemic had an enormous medical, societal, and economic cost, and was associated with previously poorly characterized disease manifestations including congenital Zika syndrome and Guillain Barré syndrome. The repercussions of this outbreak are still felt today, with communities and healthcare systems continuing to bear the burden of its long-term consequences. During that epidemic, researchers confirmed earlier reports^1,7^ that ZIKV could be sexually transmitted, as well as cause chronic inflammation in the male reproductive tract^12^. Animal model experiments have implicated the testes as a site of prolonged infection and inflammation^19,20^. Although in vasectomized men, detection of ZIKV is still possible, suggesting ZIKV may infect tissues distal from the vas deferens, such as the prostate, seminal vesicles, and bulbourethral glands^32^. The testes remain as a key site of viral replication and long-term maintenance, and impact on this organ may affect fertility.

We have shown that human Sertoli cells can support high levels of ZIKV replication without resulting in cell death, in agreement with previous reports^33,34^. This suggests that Sertoli cells may play a critical role in virus persistence. In the mouse testicular cells 15P-1, which exhibit characteristics of Sertoli cells, we also detected ZIKV replication, although at much lower levels than in the human cells. The reason for that low replication in 15P-1 is unknown, but it could reflect the fact that these cells originate from mice that do not have interferon deficiency.

For other viral infections of the testes, such as mumps virus, cytokines play a role disrupting the BTB for the virus to first enter the testicular (ST) environment^35^. At three weeks post-infection, we found that IL-6 and G-CSF cytokines were upregulated in the plasma (Fig. 3A). Higher IL-6 has been associated with severity of COVID-19 (coronavirus disease 2019) in humans^36^. It is possible that earlier in infection, more cytokines were upregulated in the plasma, leading to a breakdown of the BTB that may accelerate ZIKV entrance in the testicular environment. Cytokines known to disrupt the BTB integrity include IL-6^37^, which was detected in plasma of the A129 mice three weeks after initial infection (Fig. 3A), TNF^38^, IL-1^39^, and TGF-β3^38^. Some cytokines are also able to cross the BTB, such as G-CSF^40^, which was also detected (Fig. 3A). However, since ZIKV can directly infect Sertoli cells (Fig. 1), it may be able to partly bypass the BTB. In the testes, we found that high levels of chemokines CCL-3, CCL-4 and CCL-5 were present, suggesting recruitment of immune cells and chronic inflammation. Several cytokines were also increased over 10-fold in individual infected mice in comparison to uninfected controls, but when data from multiple animals was pooled for statistical analysis, it did not reach significance.

In a ZIKV infection model using A129 mice we only detected CD8^+^ T cells at the earlier timepoint of 7 dpi. However, it is possible that myeloid cells had already infiltrated the tissue at an even earlier timepoint but were no longer present at 7 dpi. Some myeloid cells, such as macrophages and dendritic cells have been shown to be particularly important in early infection for ZIKV to establish testicular infection^26,29^. We found the presence of myeloid cells, CD4^+^ T cells and CD8^+^ T cells in the testes at 23 dpi. This contrasted with the lymphoid organs, which had an increase in most of the immune cells at 7 dpi, but the levels of these cells then decreased or stayed similar at 23 dpi, possibly reflecting the systemic viral clearance at that timepoint. We treated A129 or C57BL/6 J mice (transiently depleted for IFN signaling using antibodies) with anti-CD3, with the purpose of depleting CD4^+^ and CD8^+^ T cell populations and function^41^. We did not observe statistically significant differences between control and CD3-depleted mice in testicular weight, histological assessments, or viral load in the testes. This was in strong contrast to the experiment assessing C57BL6/J CD8^−/−^ mice, which completely lack CD8^+^ T cells and had increased testicular damage. The discrepancy may arise from anti-CD3 treatments depleting not only CD8^+^ T cells but also CD4^+^ T cells and other cell types^42^. It is possible these cells contribute to exacerbated immune response leading to more testicular damage, and that, conversely, some reduction in their population improves the immune response profiles and mitigates testicular damage, counteracting the effects of CD8^+^ T cell depletion. Alternative explanations for these differences may be that some presence of CD8^+^ T cells is required to avoid severe testicular damage, but that having reduced levels of CD8^+^ T cells may not present as a particular issue, or due to differences in the regimen of anti-IFN antibodies in between these two experiments. Studies to separate these possibilities and investigate the role of other cells of the immune system seem warranted.

In agreement with previous reports that CD8^+^ T cells are protective in the context of systemic infections^43^, we show these cells are also important in the context of testicular infection, for both viral clearance and reduced chances of testicular damage. A similar protective role for CD8^+^ T cells has been shown in the context of the central nervous system, which like the testes is immune privileged^31^. CD8^+^ T cells recruited in a CXCR3-dependent manner can effectively control the virus in the brain if they arrive early after the viral invasion in sufficient numbers and appropriate differentiation state^31^.

A limitation of this study involves the use of mouse models, in which testicular damage appears to be more severe than that observed in non-human primate models. However, our results in these mouse models are consistent with human and non-human primate data showing persistent viral shedding and inflammation in the male reproductive system. Therefore, we posit that our results may offer mechanistic insights relevant to humans. These results may also imply that individuals who are immunocompromised, especially those with conditions known to impair CD8^+^ T cell function, should exercise additional precautions to prevent ZIKV infection.

In summary, our results offer insights into the kinetics of immune cell infiltration in the testes and the role of CD8^+^ T cells in testicular damage in the context of long-term ZIKV testicular infections. We observed early infiltration of CD8^+^ T cells in the testes, and the presence of CD8^+^ T cells promote viral clearance and reduce chances of testicular damage.

## Methods

### Cells and viruses

Vero CCL-81 and 15P-1 cells (American Type Culture Collection) were grown in Dulbecco’s minimal essential media (DMEM, Gibco, Thermo Fisher Scientific) with 10% fetal bovine serum, 200 U/mL penicillin and 200 mg/mL streptomycin (DMEM, Gibco, Thermo Fisher Scientific). hpSertoli cells were purchased from and grown in Sertoli cell growth media (IX Cells Biotechnologies) supplemented with 10% fetal bovine serum (IX Cells Biotechnologies), Sertoli cell growth supplement according to the manufacturer’s recommendations (IX Cells Biotechnologies), and antibiotic-antimycotic according to the manufacturer’s recommendations (IX Cells Biotechnologies). Sertoli cells were used in passage 3 after acquisition. ZIKV PRVABC59 was received from the World Reference Center for Emerging Viruses and Arboviruses (WRCEVA), UTMB at Vero passage 4. The virus underwent two additional Vero cell passages to generate the stocks utilized in these studies, as previously described^44^.

### Virus titration by plaque assays

Plaque assays were performed on Vero monolayers on 12-well plates as previously described^19^. Briefly, samples were diluted serially and used to infect Vero monolayers and after 1 h rocking at 37 °C, overlayed with DMEM containing 0.8% methylcellulose. Plates were incubated at 37 °C in an atmosphere with 5% CO_2_ for approximately 108 h before fixation with a 10% formaldehyde solution. The cells were fixed with a solution of 0.2% crystal violet in 30% methanol to visualize the plaques. Data are shown as PFU/ml or PFU/mg, with the limit of detection (LOD) indicated on each graph by a dotted line. For PFU/mg, each sample had slightly different LODs, and the dotted line on the graph represents the highest LOD. Values below the LOD were set to half of the LOD for statistical and graphing purposes.

### Animal infections

A129 mice are maintained in sterilized caging in a breeding colony at UTMB. C57BL/6 J and CD8^−/−^ (B6.129S2-Cd8atm1Mak/J) were obtained from Jackson Laboratory. Animals were ear-notch identified. All animal manipulations were done following an approved Institutional Animal Care and Use Committee (IACUC). The animal weights were taken up to 14 dpi daily and every third day thereafter. 9–10-week-old A129 mice were infected intraperitoneally with a target dose of 3 log_10_ PFU/mouse. 8-9-week-old C57BL/6 J and CD8^−/−^ were infected with a target dose of 5 log_10_ PFU/mouse. C57BL/6 J and their CD8^−/−^ counterparts were treated with 1.5 mg/mouse IFNAR-blockading antibody (MAR1-5A3, Leinco) once (one day before infection, in studies shown in Supplementary Fig. 4) or twice (one day before infection and 5 dpi, in the study shown in Fig. 7). In the experiment shown in Fig. 1, 5 mice were used as PBS controls, and 5 mice were used in the infection experimental condition. In the experiment shown in Figs. 2 and 3, 5 A129 mice were used as PBS controls and 6 mice were used for the infected condition. In the experiment shown in Figs. 4, 5, and 6, 3 mice were used in the PBS condition and 5 in the ZIKV-infected condition. In the experiments shown in Fig. 7, 5 C57BL/6 J mice were used in each condition, and 10 A129 were used in each condition. In the experiment shown in Fig. 7, 3 WT and 3 CD8^−/−^ were used in the PBS conditions; 10 WT and 10 CD8^−/−^ were used in the infected groups. In the experiments shown in Supplementary Fig. 4, A129 or C57BL/6 J mice were used. Two experiments were done with A129 mice, and the data combined, and one with C57BL/6 J, each experiment using 5 mice per group. For experiment shown in Supplementary Fig. 4, 0.1 mg/mouse of anti-mouse CD3ε F(ab’)2 fragments (clone 145-2C11, BioXCell) or control f(ab’)2 fragments of polyclonal hamster IgG (BioXCell) were injected intraperitoneally on days -3, -1, 2, 7, and 14 relative to infection at day 0.

### Multiplex cytokine/chemokine assay

To measure levels of cytokines and chemokines in the plasma and the testes macerates of mice, the samples were prepared as instructed by the manufacturer and read using Bio-Plex Pro Mouse Cytokine 23-Plex (Bio-Rad). Samples were run in technical duplicates, and the average was used.

### Flow cytometry

To quantify testicular cell populations, individual testes were macerated through 70-µm cell strainers and digested with 0.05% collagenase type IV (Thermo Fisher Scientific) in RPMI 1640 medium for 30 mins at 37 °C. These samples were again passed through 70-µm cell strainers to prepare single-cell suspension. Samples of inguinal lymph nodes, mesenteric lymph nodes, and the spleen were passed through cell strainers in RPMI 1640 medium to prepare single-cell suspensions. Red blood cells were removed using red blood cell lysis buffer (Sigma Aldrich) according to the manufacturer’s instructions. For surface marker analysis, leukocytes were treated with FcγR blocker CD16/32 (2.4G2, BD) and then incubated with fluorochrome-labeled antibodies or viability dye for 30 min. For analysis of intracellular markers, cells were stimulated with either ZIKV peptide (Env294–302, 5 ug/ml) or PMA (50 ng/ml)/ionomycin (750 ng/ml), in both cases with the addition of brefeldin, for 5 h, followed by the fixation and permeabilization using IC buffer (Thermo Fisher Scientific). For analysis of CD8^+^ IFN-γ^+^ or CD8^+^ TNF^+^ T cells, cells were stimulated with ZIKV peptide and for analysis of CD4^+^ IFN-γ^+^ or CD4^+^ IL-17^+^ T cells, cells were stimulated with PMA/Ionomycin. The fluorochrome-labeled antibodies used were purchased from Thermo Fisher Scientific, Tonbo Biosciences or Biolegend as below: Live Dead Fixable Dye (eFluor 506), PE-Cy7 anti-CD3 (clone 145-2C11), violetFluor 450 anti-CD8 (Clone 53-6.7), PerCP-Cy5.5 anti-CD4 (clone RM4-5), FITC anti-CD4 (Clone GK1.5), BV711 anti-CD44 (IM7), APC anti-CD62L (Clone MEL-14), FITC anti-CXCR3 (Clone CXCR3-173), PerCP-Cy5.5 anti-CD11b (Clone M1/70), BV421 anti-F4/80 (Clone T45-2342), APC anti-Ly6G (Clone 1A8), FITC anti-CD11c (Clone N418), APC anti-IFN-γ (Clone XMG1.2), PE anti-PD-1 (clone J43), PerCP-eflour 710 anti-TNF (Clone MP6-XT22), and PE anti-IL-17 (Clone ebio17b7). To inactivate the virus, cells were fixed in paraformaldehyde 2% for 16 h at 4 °C. Cell samples were acquired on BD LSR Fortessa and data were analyzed (Supplementary Fig. 1) using FlowJo 10 (BD).

#### Histology

Samples of tissue were collected and placed into a solution of 10% neutral buffered formalin (Thermo Fisher Scientific) for 24 h. Following this incubation, the formalin solution was replaced with 95% ethanol^45^ and sent to UTMB’s Anatomic Pathology Laboratory core facility, where samples were paraffin embedded, and either stained with hematoxylin-eosin (H&E) or left unstained and used for immunofluorescence analyses. A blinded board-certified pathologist read the H&E slides.

### Immunofluorescence assay

To conduct immunofluorescence assays, a modified protocol from a previous study was used^46^. Sections of mouse testes on slides were dewaxed by heating at 65 °C overnight, followed by incubations in xylene and graded ethanol series (Millipore, Sigma). Antigen retrieval was performed by incubation in citrate buffer pH 6 (Abcam) at 90 °C for 30 min. Sections were then washed in deionized water and blocked with a solution of 5% normal goat serum (NGS) in PBS for 15 min at 37 °C and then stained overnight in a humidified chamber at 4 °C with 4G2 antibody (Abcam) at 1:200 and DDX4 antibody (Abcam) at 1:500 diluted in 5% NGS. Washed in PBS and incubated with 0.2% Sudan black B diluted in 70% ethanol for 12 min at 37 °C in a humidified chamber. The sections were then incubated for 45 min at 37 °C with a secondary antibody at a dilution of 1:2000, AlexaFluor 594 anti-human IgG (Thermo Fisher). The slides were mounted with ProLong Gold Antifade Mountant with DAPI (Thermo Fisher). Images were captured using an inverted fluorescence microscope (Olympus-IX73), and brightness and contrast were adjusted in all images equally to ensure optimal visibility.

### Quantification and statistical analysis

Mouse groups’ weight data were compared at each time point using two-way ANOVA with Tukey’s multiple comparison correction. For comparing ZIKV positive/negative immunofluorescence of the testes, Fisher’s exact test was used. For all other statistical tests, either one-way ANOVA with Sidak’s multiple comparison testing was used for comparison between multiple groups or two-tailed student’s t-test was used when only two groups were being compared. Viral titers were log_10_ transformed before statistical tests were applied. Left and right testicular weights and percent of spermatogenesis were averaged before statistical analysis. In the experiment presented in Supplementary Fig. 4, only the left testis data were available because the right testis was used for titration data, and cutting the testes was found to reduce the quality of the histology data. Data from flow cytometry, BioPlex, testicular weight, and percent spermatogenesis were compared using one-way ANOVA with Sidak’s multiple comparison testing. For all analyses, a p-value lower than 0.05 was considered significant. Statistical analyses were done using the software GraphPad version 8.4.3.

## Supplementary information

Supplementary Materials

## Data Availability

The datasets used and/or analyzed during the current study available from the corresponding author on reasonable request.
