# Supplementary material for: CD8+ T cells promote ZIKV clearance and mitigate testicular damage in mice
Source: Npj Viruses. 2024 Jun 17;2:20. doi: 10.1038/s44298-024-00033-5 (PMC11721072; doi:10.1038/s44298-024-00033-5)
Supplement: Supplementary file 1 — Supplementary Materials [file 44298_2024_33_MOESM1_ESM.pdf]

SUPPLEMENTARY MATERIALS

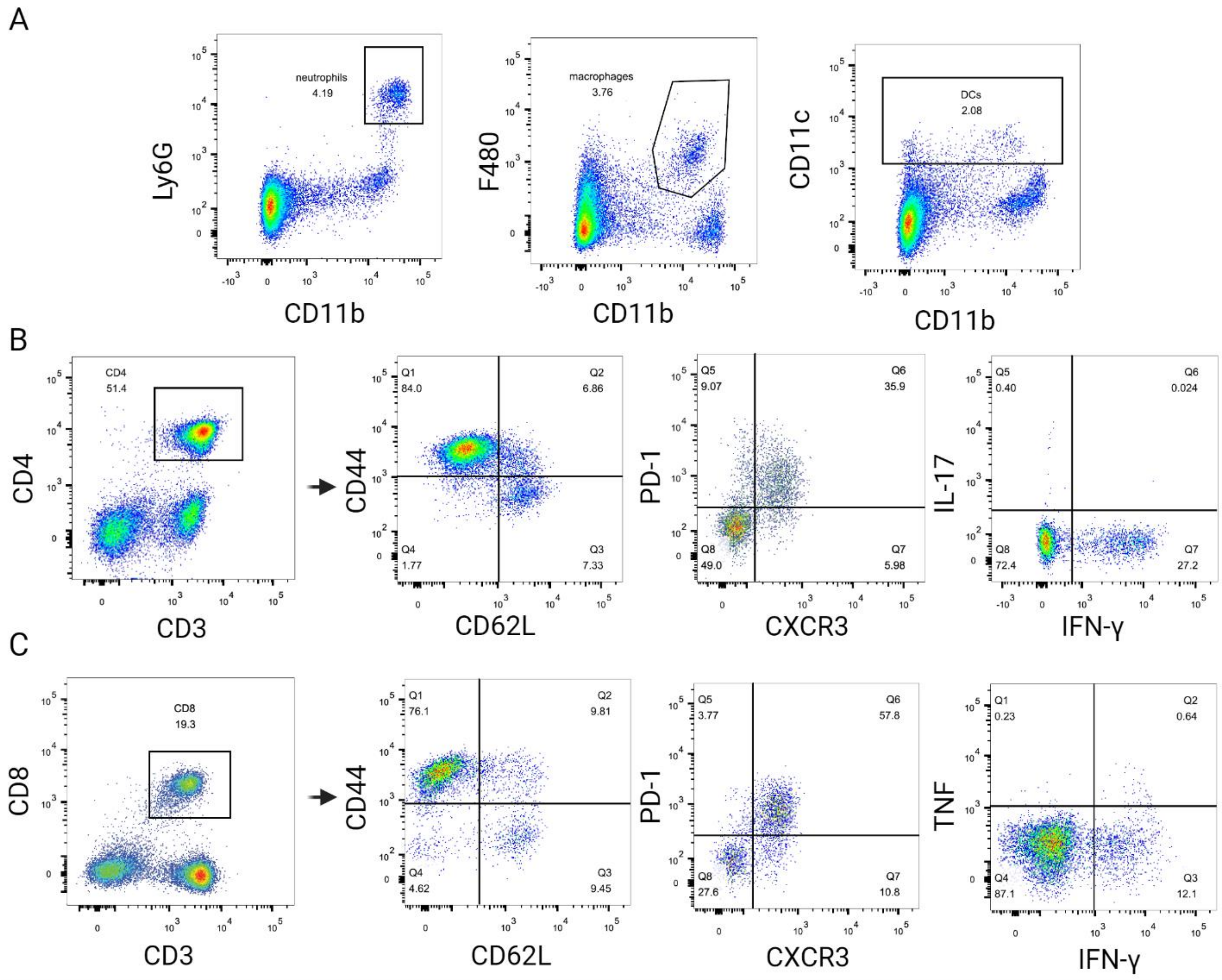

Supplementary Figure 1. Flow cytometry gating strategy. Cells were first gated by side scatter and forward scatter, singlets were selected, live/dead-negative cells were selected. A. Neutrophils (Ly6G$^+$ CD11b$^+$), macrophages (F480$^+$ CD11b$^+$) and dendritic cells (CD11c$^+$) gating strategy. B. CD4$^+$ T cells gating strategy. These cells were then selected and gated for effector status (CD44^{hi} CD62L^{lo}), PD-1, CXCR3, IL-17 and IFN-gamma. C. CD8$^+$ T cells gating strategy. These cells were then selected and gated for effector status (CD44^{hi} CD62L^{lo}), PD-1, CXCR3, TNF and IFN-γ.

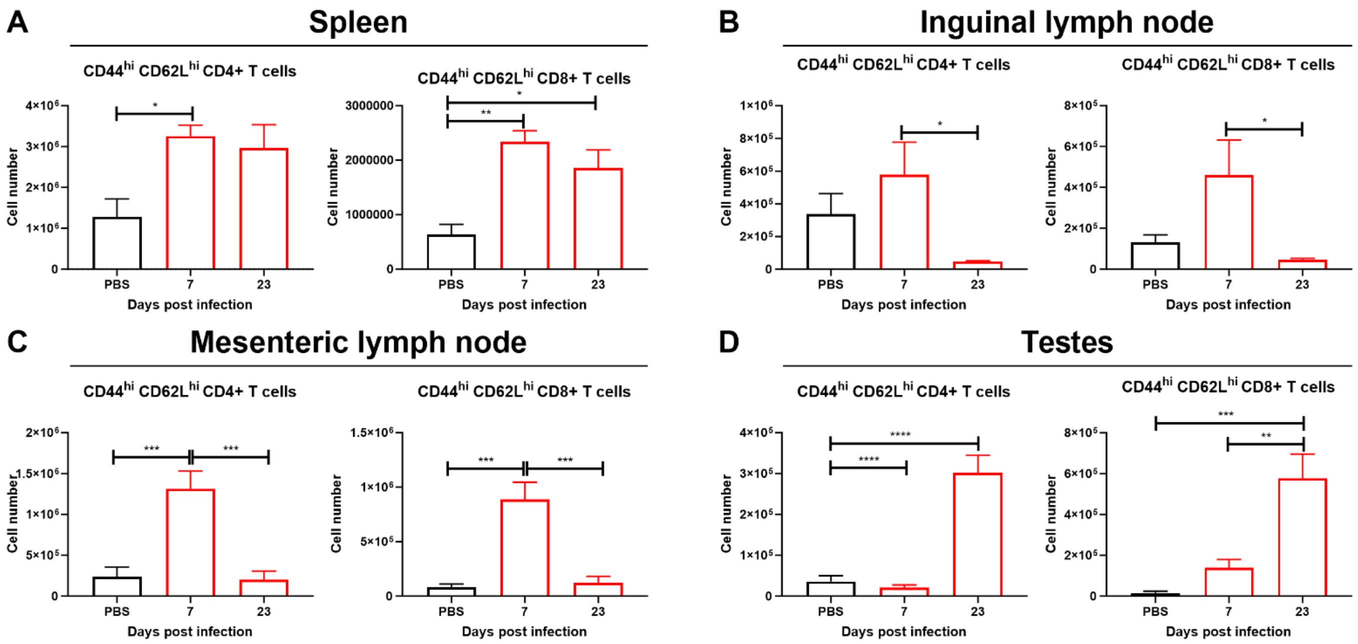

Supplementary Figure 2: CD44^{hi} CD62L^{hi} T cells are present in testes at 23 dpi. Possible memory CD8^{+} and CD4^{+} T cells detected in A. the spleen, B. the inguinal lymph nodes, C. the mesenteric lymph nodes, D. the testes. Red bars show ZIKV-infected mice, black bars denote uninfected baseline controls. The data shown in this figure are from the same mouse groups as in Figure 4. Statistical significance was assessed with one-way ANOVA with Sidak's multiple comparison correction. Comparisons were made in between each of the groups and only significant results are shown. Bars show mean and error bars represent standard error of the mean. *, $p < 0.05$; **, $p < 0.01$; ***, $p < 0.001$; ****, $p < 0.0001$.

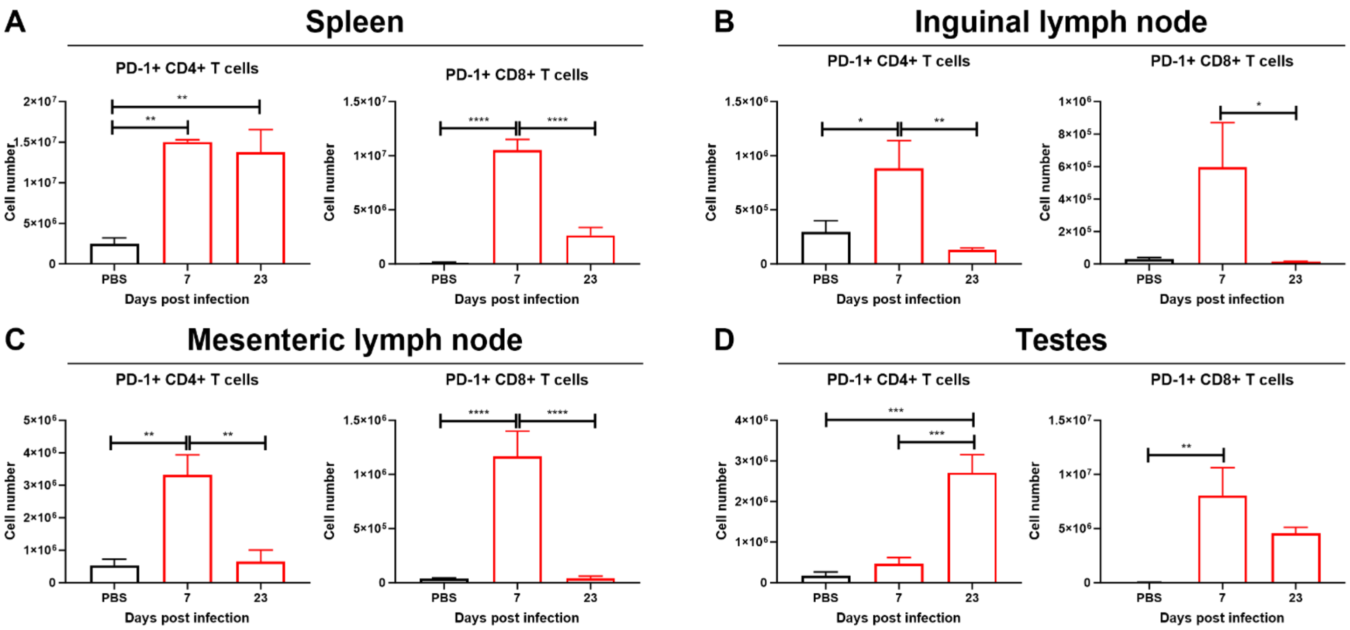

Supplementary Figure 3. Analysis of PD-1$^+$ T cells. Cell number of PD-1+ T cells in A. the spleen, B. the inguinal lymph nodes, C. the mesenteric lymph nodes, D. the testes. Red bars show ZIKV-infected mice, black bars denote uninfected baseline controls. The data shown in this figure are from the same mouse groups as in Figure 4. Statistical significance was assessed with one-way ANOVA with Sidak's multiple comparison correction. Comparisons were made in between each of the groups and only significant results are shown. Bars show mean and error bars represent standard error of the mean. *, $p < 0.05$; **, $p < 0.01$; ***, $p < 0.001$; ****, $p < 0.0001$.

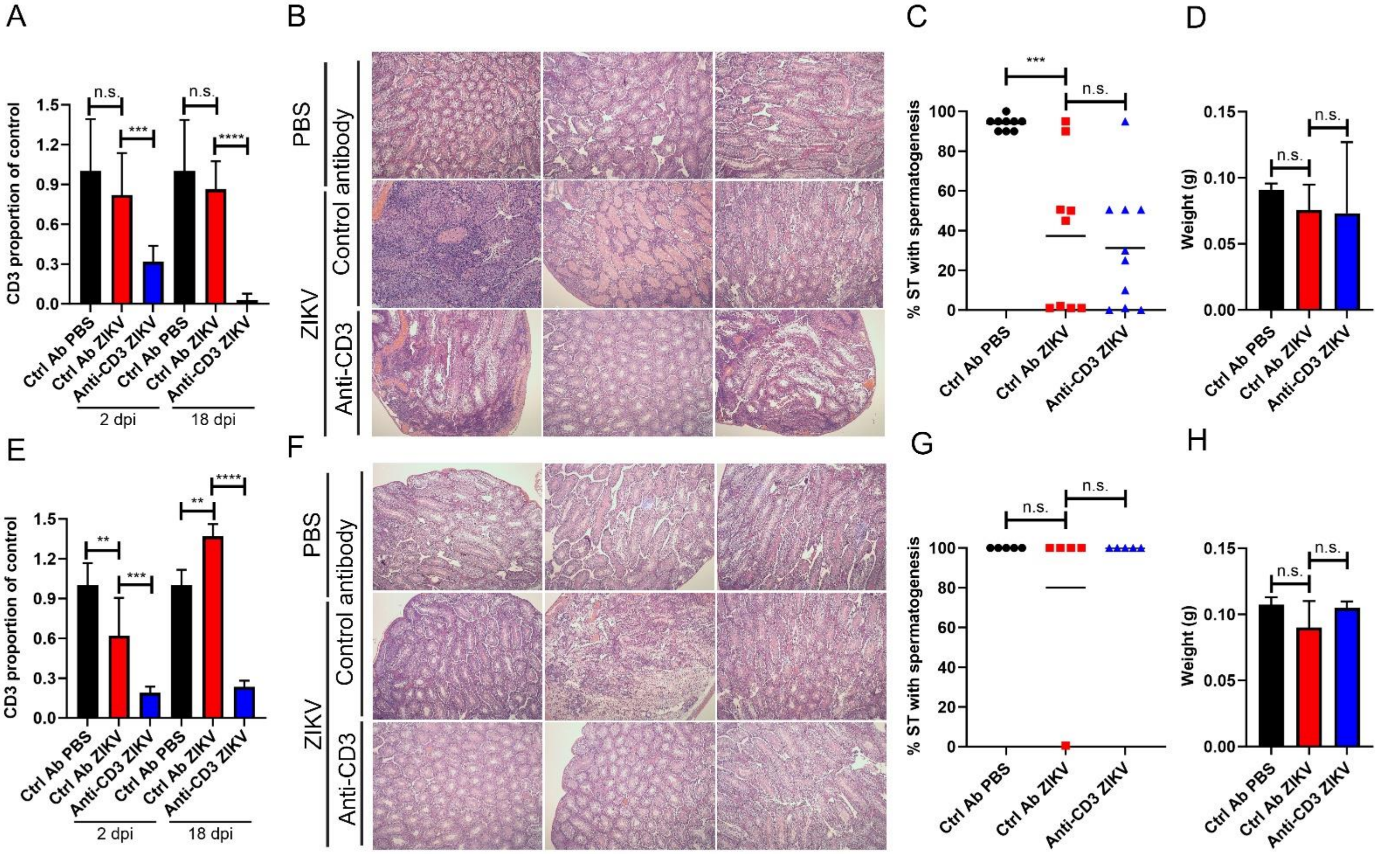

Supplementary Figure 4: Depletion of CD3 did not significantly impact mice testicular damage. A. Proportion of CD3 events in blood samples of A129 mice excluding red blood cells at 2 or 18 dpi. A129 mice were infected intraperitoneally with a target dose of $3\log_{10}$ PFU/mouse. These data points are combined from two experiments, with a total of 9 mice in the control antibody PBS group, 9 mice in the control antibody ZIKV group, and 10 mice in the anti-CD3 ZIKV group. B. Histology of A129 mice testes. C. Percentage of seminiferous tubules (ST) with spermatogenesis in each A129 mouse testicular sample. D. Testicular weight of A129 mice. E-F show proportion of CD3 events, histology, percentage of ST with spermatogenesis, and testicular weight of C57BL/6J mice. C57BL/6J mice were infected intraperitoneally with a target dose of $5\log_{10}$ PFU/mouse. In each group, 5 C57BL/6J mice were used. Statistical significance was assessed with one-way ANOVA with Sidak's multiple comparison correction. Bars show mean and error bars represent standard deviation. Lines represent the mean. **, $p < 0.01$; ***, $p < 0.001$; ****, $p < 0.0001$; n.s., no significant difference.

Supplementary Table. Mice with testicular samples positive for ZIKV Envelope protein (4G2 antibody)

Mouse	# mice with negative testes	# mice with at least one positive testis
WT	10	0
$CD8^{-/-}$	4	6

Fisher's exact test p-value = 0.01
